# Cases of Injuries of the Head, Treated at the Middlesex Hospital

**Published:** 1827-01

**Authors:** 


### INJURIES OF THE HEAD.

### *Cases of Injuries of the Head, treated at the* Middlesex Hospital.

Wounds of the head derive their importance from their connexion with, or from their proximity to, the brain: the following series of cases will therefore commence with the most simple injuries, viz. those of the scalp.

I. *Case of a Lacerated Wound of the Scalp, which was followed by Erysipelas, and by the Death of a Portion of the Skull.* Treated by Mr. ~~Joberns.~~ Shaw

Daniel Maynard, ætatis sixty-three, a fine healthy looking old man, was brought to the hospital on the evening of the 7th of November, 1825, with an extensive laceration of the scalp. He said that he had been driving a hackney-coach; that the box broke down, and that he fell forwards on his horses, and from thence to the ground. The horses were frightened by his struggles to extricate himself, and set off at full speed: he still grasped the reins and held the whip, so that he was dragged a considerable distance with great rapidity. He was picked up in an almost insensible state, and conveyed to a surgeon. When he had somewhat recovered his senses, he was brought to the hospital.

The wound of the scalp commenced just above the right ear; it extended to the external angular process of the frontal bone on the same side, and then took a direction upwards and backwards, and terminated at the vertex. Thus a large flap was formed, which was reflected upon the occiput: this flap-like portion of scalp was much bruised, and on either side was covered with mud. The pericranium was torn off extensively, leaving a large portion of denuded bone. There was a small and inconsiderable wound at the back part of the head.

The head was shaved; the loose and ragged portions of the pericranium were removed; and the flap of the integuments was made perfectly clean. It was then replaced, and retained in its situation by adhesive straps and a roller lightly applied. He took a dose of Calomel and Jalap, which procured free evacuations from the bowels.

On the following morning, the pulse was quick and small; the tongue was white and moist, and the skin dry. The bandage was removed: the edges of the wound were observed to be red, and surrounded by a blush of inflammation; the wound appeared to be in a state approaching to suppuration.

He was ordered to take the following draught three times a-day:—Haustus Salini ℥ ij.; Vin. Antim. T., Spirit. Æther. Sulph. C. āā m. xxv. And a pill containing Calomel gr. iij. Opii gr. j. at bed-time.—Low diet.

10th.—He remained much in the same state as regarded the general symptoms. The pulse was feeble, and the tongue clean; the wound was suppurating, and discharged a healthy pus, but its edges were far separated. An erysipelatous inflammation had spread from the wound, and extended down the neck.

As the pulse had diminished both in frequency and strength since yesterday, the saline mixture was omitted.—Inf. Gentianæ C. ℥ jss.; Træ. Gent. C. ʒ j. M. fiat haust. ter in die sumend.—Vin. rubri ℥ ij. quotidie.—He was allowed a more generous diet.

12th.—The erysipelas had somewhat diminished. He complained of no pain in the head, but merely of soreness in the wound, the edges of which had retracted, and exposed parts of the parietal bone denuded of its periosteum. Pulse still small and weak.

Vin. rubri ℥ iv. quotidie.—The bowels being confined, some aperient medicine was prescribed.

16th.—The wound was now suppurating kindly, but its edges had receded still farther, and left uncovered a portion of bone about nine or ten inches in circumference. The erysipelas had declined.

Inf. Cinchonæ ℥ jss.; Træ. Cinchonæ C. ʒ j. ter die sumend.

21st.—Appeared to be improving. The granulations were shooting up, and looking very healthy. The exposed part of the bone was of a whitish-grey colour, and seemed to have lost its vitality.

He continued to take his wine and bark; and occasionally, when any symptoms of irritation arose, he took Calomel gr. iij.; Opii gr. j. at bed-time.

December 5th.—So favourable had been the progress of this case, that it was thought unnecessary to lengthen it out by a daily report. To-day, however, he appears dull; his temper is easily ruffled, and he is inclined to be sulky. He refuses his medicines, but promises to take them at night. The wound no longer secretes a bland and healthy pus; its surface is dry, and a sanious discharge oozes from it. The tongue is dry, and covered with a yellowish fur.

Three grains of Calomel and three grains of Antimonial Powder to be taken at night.

6th.—He is delirious, and has been so during the whole of the night. Tongue brown and dry; skin hot, and without perspiration; pulse weak and fluttering. He much resembles a patient in the last stage of continued fever.

Haust. Salin. ℥ ij.; Vin. Antim. T. m. xx. sextis horis sumend.—The bark to be discontinued.

Towards evening, he fell into a comatose state, and died during the night.

*Dissection.*—The pericranium surrounding the dead portions of bone, as seen externally, was much thickened, and, on being separated from the living bone, the latter presented a worm-eaten appearance: it was perforated by numerous minute holes, which seemed to have been caused by a sort of interstitial absorption, which had been going on in the substance of the bone. The same appearance presented itself on the internal surface of the skull. A small portion of bone, far removed from the centre of the external caries, was diseased throughout its whole thickness. The dura mater at this part had separated from the bone, and was thickened; the surface of the brain immediately beneath was not discoloured. The whole mass of the brain appeared to have been in a state of general excitement, for an effusion of serum into the ventricles had taken place: these were much enlarged. No obvious appearances of inflammation were present.

The operation of trepan, in such a case as the above, becomes a most difficult question. It is a most dangerous proceeding to cut away so large a portion of the skull. The circumstances of the case should be well weighed in the mind of the surgeon. It is an established rule that, if the operation is to be performed, it must be done before the symptoms of excitement come on, or after they have subsided.

II. *Case in which the Scalp and Pericranium were destroyed by a Burn, and the Bone rendered Carious.* Treated by Mr. Shaw.

March 22d, 1826.— —— Henderson, aged thirty-seven. The anterior part of the right parietal, together with a portion of the frontal bones, having the coronal suture passing through the centre, are denuded of their scalp and periosteum; the surface has lost all the florid appearance of living bone; it is dry and of an ash-colour, but here and there it is beginning to put on the darker hue of caries. A fissure, sufficiently large to admit the rounded extremity of a common probe, marks the recession of the living from the dead bone. The right eminentia frontalis is similarly diseased; and the whole is surrounded by an elevated line of dark-coloured unhealthy granulations:

It appears that this man has been subject to epileptic fits for the last twenty years: these fits occurred at irregular periods, the intervals between which were seldom prolonged beyond eight weeks. About ten weeks ago, while sitting alone and near the fire, he fell from his chair in a fit, and his head came in contact with the heated bars of the grate: a severe burn of the scalp, covering a considerable portion of the right parietal and a part of the frontal bones, was the result. About four or five days after this accident, the injured parts sloughed, and the bone was exposed. He had now for the first time an attack of maniacal delirium, which lasted about two days; and he has since had three similar paroxysms. It is stated that, on the decline of these, he has usually fallen into a profound sleep, from which, after some hours, he has awoke perfectly recovered. According to his friends, these attacks have observed regular intervals of three weeks. He has a slight impediment in his speech, but, on enquiry, it was found that he has always been subject to this. His general health did not seem to be affected until about three weeks since; his strength then began to diminish, since which time he has become gradually weaker, and he is now unable to walk without assistance. His intellects have suffered, his memory fails him, but still his answers to questions are perfectly rational. He does not complain of any pain in his head, except soreness of the wound. The pulse is seventy-five, and natural; tongue clean.

He is to take at bed-time, of Calomel gr. iv.; Jalapæ gr. viij.; Antimon. Powder gr. iij.—The bowels are to be kept moderately lax with the compound Senna Mixture; and he is to take a Saline Mixture, with small doses of the Tartarised Antimony, three times a-day.

29th.—No alteration in his symptoms occurred before this morning, when his periodical attack of mania returned, but not with more than its usual violence, although so severe as to call for the coercion of a straight-waistcoat. Pulse ninety, and full; tongue dry and brown. His symptoms generally resemble those of a patient labouring under acute typhus.

Ten ounces of blood were taken from the temporal artery, and a blister was applied to the nape of the neck; the head is to be shaved, and a cold lotion applied.—He is to take Calomel gr. iv. Antimon. Powder gr. iij. at night; and continue the mixture as before.

31st.—He is again becoming rational, and answers questions. The muscles of the face corresponding with the affected side of the head are partially paralysed.

April 3d.—He became quite paralytic on the morning of the 1st, and in the afternoon gradually fell into a comatose state. He remained in this condition until his death, which took place last night. The surface of the body was covered with petechiæ.

*Dissection.*—The skull-cap being removed, it was found that a part of the dura mater, not much larger than a shilling, and situated under the larger portion of diseased bone, was detached from the skull, and thinly covered with an extremely minute quantity of a sero-purulent fluid: lymph had been thrown out upon the surface, so as to produce some degree of inequality and thickening in this part of membrane. A small shred of lymph was found attached to the inner and secreting surface; the other parts of this membrane were healthy. No thickening of the arachnoid membrane, or other signs of inflammation, were observable, except about an ounce of limpid serum contained in the ventricles. In other respects the brain seemed perfectly healthy, and retained its natural degree of firmness throughout.

It does not appear that this man had at any period symptoms which indicated the propriety of an operation: he had none of those which occur from the presence of dead bone; none of those which are so characteristic of the inflamed dura mater. Nor can we revert, with a better prospect of success, to the old theory of compression, in which the minutest quantity of pus was gifted with so much importance. The maniacal delirium occurred, most probably, before the bone was dead throughout its substance; and again, it entirely subsided without the removal of the cause, supposing it to have depended upon the caries. These circumstances recall to our recollection the observation of Dr. Prichard, that, "in very severe and inveterate cases of epilepsy, the paroxysms of this disease are often followed by attacks of maniacal delirium, which are generally of a most violent kind. These fits most commonly abate in a few days after the epileptic attacks have ceased."*

In considering the circumstances of the case, it must be remembered that the patient had been for many years the subject of severe epilepsy; and that he had a large portion of the skull deadened by an agent the most destructive and immediate in its influence on living parts. The disposition of the living bone to recede from the dead became most important, as indicating the possibility of two occurrences: first, that the disease extended no further than the deploe, and that an exfoliation might take place; secondly, it held out a prospect that both tables might be thrown off by a natural process, should the disease have extended through them; as in the cases related by Mr. Pott and Mr. Abernethy.

### CONCUSSION.

The following cases are good examples of various degrees of concussion, with their consequences.

### III. *Case of Concussion, followed by Inflammation of the Brain.* Treated by Mr. Bell.

William Spirit, ætatis twenty-four, was admitted October 10th, 1826. Four days before his application at the hospital, he had

* Prichard on Nervous Diseases, p. 62.

fallen on some pavement from a hayloft, a height of fifteen feet: he was stunned by the fall, and remained insensible for a few minutes. He shortly afterwards felt some pain in his head, which had been gradually increasing up to the time of his admission.

The pain in the head was now exceedingly acute: it was confined principally to the occiput and forehead; he occasionally felt a pain shoot through the head from temple to temple. The temperature of the scalp was increased; there was great intolerance of light. He had a dull and heavy look, —an expression of countenance very remarkable in some forms of continued fever: the eye presented that appearance which some have described as an *intellectual dulness* combined with *physical brightness;* the upper eyelid dropped over the eyeball. The pulse was about ninety, quick and hard, but rather weak; the bowels were open; the tongue was dry and furred. The symptoms altogether were so like those of continued fever, that a person, ignorant of the previous history of the case, would have declared it to have been a case of fever. There had been a small and superficial wound on the occiput, which was nearly healed.

The head was shaved, and a cold lotion applied. He was well purged with Calomel and Jalap.

11th.—The symptoms were somewhat relieved: the tongue was moist and less furred; pulse continued much the same as before.

Sixteen ounces of blood were taken from the back of the neck by cupping. —Haust. Salin. ℥ ij.; Vin. Antim. Tart. ʒ j. ter in die sumend.

16th.—His head was much relieved by the cupping. His pulse retained much of its sharpness, but it was compressible; his tongue was clean and moist. The wound of the scalp was perfectly healed.

Hirudines ℥ xij. pone aures applic.—To continue the antimonial mixture.

He lay for some days in a dull, heavy, and stupid state. By a perseverance in the local depletion, the pain in the head gradually abated; and, in proportion as this took place, in the same proportion were the natural functions of the brain restored. He was discharged October 24th. There was still to be observed a want of "speculation in his eye," and although he was free from pain in the head, yet this appearance seemed to show that the disease was not completely subdued. He left the hospital with directions to continue the antimonial mixture, to live low, and remain perfectly quiet for some days.

October 26th, he again made application for admission. The pain in the head returned soon after he left the hospital. He stated that, on the day following, the noise of the carriages rang in his ears, and at length became very distressing to him. Walking now gave him pain in his head: this was greatly aggravated on coming down stairs. When desired to stamp on the floor, the pain in the head was much increased. The light was painful to him; pulse eighty-eight, quick, and rather hard.

Nauseating doses of Tartarised Antimony were again prescribed, and leeches were applied to the temples; the nape of the neck was blistered; and he was frequently purged with Calomel and saline aperients.

Under this plan of treatment, all the unfavourable symptoms were removed. He regained his wonted expression of countenance, (which, by the by, appears never to have been endowed with much vivacity;) and he was discharged on the 7th November, being at that time perfectly convalescent.

### IV. *Case of Concussion, followed by Symptoms of Inflammation of the Brain.* Treated by Mr. Shaw.

William Todd, ætatis thirty-four, a carpenter, a man of spare habit, and of the melancholic temperament, fell a perpendicular height of nine feet, upon a stone floor; and he says that he struck upon his head. However this may be, he remained perfectly insensible for twelve hours. When he had recovered so far as to recognise those around him, he began to complain of a dull and aching pain in his head: this increased, and on the second day after the accident he came to the hospital, and one of the dressers cupped him at the back of the neck, abstracting twelve ounces of blood.

He was brought here on the 23d October, six days after the fall, and was admitted. He had great pain in the head, which was constant. His rest had been disturbed: indeed, he stated that he had not slept for five nights or days. The pain occasionally darted through the head. There had been no remission of the pain in the head since the accident: on the contrary, it had daily increased in severity. There was increased heat of scalp, and much intolerance of light; the eye presented the appearance described in the last case, only that the *intellectual dulness* was greater than in the preceding. The upper eyelid nearly covered the eyeball. This falling of the eyelid, and inability to raise it completely, was not wholly dependent upon the dislike to light; for, when the room was darkened, the heaviness of the eyelid still remained. He lay in a half comatose state; he was quite rational, and had at no time been delirious. Pulse fifty-five, quick, and vibrating, but at the same time weak and compressible; tongue dry, and covered with a yellow fur. No external injury of the head could be detected.

From the long continuance of these symptoms, which appeared to be evidently those of inflammation of the brain,—from the weak and compressible pulse,—from its slowness,—and from the half comatose state in which he lay, it was considered that general blood-letting was not indicated.

Twenty leeches were applied to the temples; and he took a purge of Calomel and Jalap.

24th.—Pulse softer; pain in the head somewhat relieved. Seven ounces of blood were taken from the arm, with a view of ascertaining whether it presented the general appearances of inflammation. The blood was slightly cupped, but the buffy coat was hardly perceptible.

The head was shaved. He was directed to take Calomel gr. v. at night; and the following mixture three times a-day—Haust. Salin. ℥ ij.; Vin. Antim. Tart. ʒ j.

25th.—The symptoms were less severe, although the pain in the head was still distressing. He slept much better.

Hirudines xxiv. temporibus applic.

27th.—The improvement in this man's condition was very striking. His rest at night was now undisturbed. Eighteen leeches were applied to his temples yesterday, since which time his head has been almost free from pain.

29th.—A slight increase of pain in the head, which yesterday had been altogether absent. Pulse was harder, stronger, and more frequent than on the preceding day; although even now it did not exceed in frequency the standard of health. Bowels confined.

The house medicine was taken every two hours, in divided doses, until the desired effect was produced; and eighteen leeches were applied to the temples.

It was found necessary to continue the local depletion for some weeks. The nape of the neck was frequently blistered; after which a seton was introduced. By these means the symptoms of cerebral inflammation were removed. His head remained giddy for some time; but, as his strength returned, this disagreeable sensation wore off, and his countenance lost much of the dull and heavy cast which had characterised it throughout. He left the hospital.

There is something worthy of remark in the two cases of concussion just related,—namely, the masked appearance which inflammation of the brain is apt to put on when that organ has received a general shock. The whole brain, with its delicate system of vessels, is much injured by the concussion, so that these vessels appear to be incapable of undergoing that great increase of action which accompanies the high delirium of phrenitis. A dilated and debilitated state of the vessels of the brain is the consequence of this injury: it allows the general symptoms of inflammation of the brain to be, as it were, mixed up with those which accompany apoplexy; and, if the inflammatory symptoms run so high as to cause delirium, that delirium partakes of the nature of typhomania. Thus, in the above cases, the patients lay for some days in a half-comatose state. In the slighter injury, the pulse rose but little above the natural standard; in the more severe injury, the pulse was actually below the natural standard; and in both cases the pulse was weak and compressible.

The following case will illustrate this point: it will show the nature of the injury which the brain suffers in concussion; and that, when the patient dies from its effects, he dies truly apoplectic.

V. *Fatal Case of Concussion of the Brain.* Under the care of Mr. Bell.

Richard Bainbridge, ætatis thirty, was brought to the hospital, ten o'clock A.M. November 6th, 1826. He had been repairing the steeple of a church; a part of the scaffolding gave way, and he was precipitated from a height of thirty feet. He was quite insensible when taken up, in which state he remained when brought to the hospital.

The breathing was laboured and stertorous; the pupils were dilated; the surface was cold, and he was pulseless; froth issued from his mouth; and, in short, he appeared to be in a dying state. There was a large and irregular wound on the vertex, and the skull was extensively fractured. The bone was broken into several pieces, and there was a small portion slightly depressed. An hour after admission, the pulse could be distinguished at the wrist: all the other symptoms continued the same. He lay in a deep lethargy, from which nothing could rouse him.

At this juncture Mr. Bell visited the patient. Mr. Bell considered that no operation could benefit the condition of the patient. He imagined that these symptoms arose from the general injury which the brain had received, and not from a local cause which was remediable by an operation.

In the evening, the pulse at the wrist became quite distinct; the heat of the surface had increased; and, on bringing a light to the eyes, the irides were observed to contract and dilate irregularly. No improvement in the other symptoms. Mustard cataplasms were applied to the feet.

7th.—We were rather surprised to find this man still alive, and precisely in the same condition as on the preceding night. He had been unable to swallow any thing since his admission; he was therefore ordered an aloes clyster, and the head was carefully shaved.

Mr. Bell saw him, and still was of opinion that this was not a case for operation; but, as no harm could accrue from the removal of the loose portions of bone, and as the danger of the patient could not be increased by such an operation, it was accordingly performed. The wound of the scalp was enlarged, and it was found necessary to set on the trephine to remove the shelving pieces of bone.* Some sensibility was manifested by the patient on the scalp being divided.

Several loose pieces of bone were now removed, so that no de-

* By the bone shelving, is meant the breaking up of the inner table of the skull to a greater extent than the outer; so that it is impossible to remove such a portion of bone without the application of the trephine, or the cranium saw.

pressed portion remained. A thin film of blood merely was found upon the dura mater. A fissure was observed extending towards the right temple, and another in the opposite direction towards the left parietal eminence. The object being accomplished,—namely, the removal of the loose portions of bone,—the wound was afterwards properly dressed; the dura mater was equably supported by pieces of oiled lint and a roller aptly applied.

Ten P.M.—The respiration continued the same; the accessory respiratory muscles were observed to be in strong action. The patient was turned upon his side, with the immediate effect of removing the stertor; but this position, by fixing one side of the chest, rendered respiration more laborious. The bowels had been opened by the glyster. The pulse was exceedingly rapid and rather hard, although by no means strong. The heart appeared to be labouring, and gave one the idea that it was oppressed with blood. He was bled to ten ounces, and the sinapisms to the feet were ordered to be continued.

He died about three o'clock on the following morning.

*Dissection.*—A fissure extended from the vertex, and ran down through the posterior part of the skull: it crossed the right parietal eminence, and reached the base of the skull, terminating within an inch of the great foramen. Another fissure ran in an opposite direction towards the right temple. A delicate film of blood was found upon the dura mater, under the line of the posterior fissure. The surface of the brain was covered with bloody spots of extravasation, presenting the appearance usually termed bloodshot: these spots were general on the exterior surface of the brain. The vessels of the brain appeared to be turgid with blood, but the minute ramifications were not injected as in inflammation. On cutting the brain, the divided vessels appeared to be enlarged, as if dilated. The structure of the brain seemed to be broken down at points; for here and there it was evidently softer than natural, and at these points extravasated spots of blood were seen. A minute quantity of blood was found in the base of the skull.*

* The first of these cases (page 15,) was treated by Mr. SHAW, not by Mr. JOBERNS. The commencement of the paper, being in the preceding half-sheet, had passed the press before we discovered the mistake. The cases are communicated by a gentleman who is not himself a surgeon of the hospital.—EDITOR.

